# Delayed active bleeding after radical evacuation of injected polyacrylamide hydrogel and immediate implant based breast reconstruction

**DOI:** 10.1186/s40064-016-3098-0

**Published:** 2016-08-30

**Authors:** Min-Ling Chen, Jin-Lan Liu, Shen-Jung Hsu, Chang-Cheng Chang

**Affiliations:** 1 Aesthetic Medical Center, Department of Surgery, Chang-Gung Memorial Hospital, Chia-Yi, No. 6 ChiaPu Road, Puzih City, Chiayi County 613 Taiwan; 2 School of medicine, College of medicine, China medical university, No.91, Hsueh-Shih Road, Taichung, 40402 Taiwan; 3 Department of Pathology, Chang-Gung Memorial Hospital, Chia-Yi, No. 6 ChiaPu Road, Puzih City, Chiayi County 613 Taiwan; 4 Department of Radiology, Chang-Gung Memorial Hospital, Chia-Yi, No. 6 ChiaPu Road, Puzih City, Chiayi County 613 Taiwan; 5 Plastic and Reconstructive Surgery, Chang Gung Memorial Hospital, Chia-Yi, No.6 ChiaPu Road, Puzih City, Chiayi County 613 Taiwan

**Keywords:** Delayed active bleeding, Polyacrylamide hydrogel, Breast reconstruction

## Abstract

**Background:**

Polyacrylamide hydrogel (PAAG) was used in breast augmentation surgery in China during 1997–2006. Its application has led to increasing complications such as localized lumps, asymmetry, diffuse stiffness, infections, and localized tenderness or myalgia. Hence, many patients have sought for surgical gel evacuation in the following years.

**Case presentation:**

A 37-year-old G2P0A2 slim (BMI = 18.3) woman who had received injected PAAG bilateral breast augmentation in China 15 years ago, visited our hospital for gel removal due to left upper quadrant breast tenderness with burning sensation. We performed simple mastectomy and immediate breast augmentation with silicone prostheses. Her postoperative course was uneventful. However, subcutaneous hematoma formed right after she started breast massage on the day 14 post operatively. The patient promptly received a CT-guided drainage followed by exploratory surgery with coagulation of the lesion located at the second intercostal space 2 cm to the right of the sternum.

**Discussion:**

Delayed bleeding in our patient might be contributed to below reasons. First, the usage of PAAG might cause degeneration of muscle tissue structure which made it more fragile than normal muscle tissue. Secondly, the lack of sufficient coverage, cushion and protection of the muscle tissue in slim patient in addition to the external compression and shearing force of massage.

**Conclusion:**

In slim patients, we suggest that the postoperative breast massage should be postponed for months until the tissue is recovered, or delayed breast reconstruction could also be considered.

## Background

Polyacrylamide hydrogel (PAAG) was thought to be a nontoxic, non-allergenic synthetic biomaterial and was applied as alloplastic soft tissue filler in cosmetic surgery in Ukraine since 1990s. It contains 2.5–5 % polyacrylamide, a synthetic polymer, and 95–97.5 % sterile water. After administrated into soft tissue, the water content will be absorbed by tissue and then the PAAG becomes encapsulated, remaining soft tactile impression (Leung et al. 2007). Because of the simple procedure, PAAG was used broadly in breast augmentation surgery in China after approved by the State Food and Drug Administration of China in 1997. However, its safety was doubtful, series of reports revealed PAAG complications such as localized lumps, migration of implants, asymmetry, diffuse stiffness, infections, localized tenderness and myalgia (Luo et al. 2011; Cheng et al. 2002; Evstatiev 2006). The application of PAAG in breast augmentation was finally banned in 2006 by the State Food and Drug Administration of China. In 2008, Cheng et al. reported two cases of breast cancer following PAAG injected breast augmentation (Cheng et al. 2009). Although no adequate evidence supports the causal relationship between PAAG injection and the development of breast cancer, it did raise the attention regarding the potential risk of carcinogenesis of PAAG.

In accordingly, there are increasing numbers of patients seeking for PAAG removal due to the above complications. Radical removal of the infiltrated fascia or simple mastectomy followed by immediate implant based reconstruction is recommended for patients with small breasts (Luo et al. 2011; Qiao et al. 2005). However, there is a lack of understanding regarding the complication of the removal of PAAG gel. This study reports a case with delayed active bleeding on day14 post-radical evacuation of PAAG followed by immediate implant based reconstruction.

## Case presentation

**Fig. 1:**
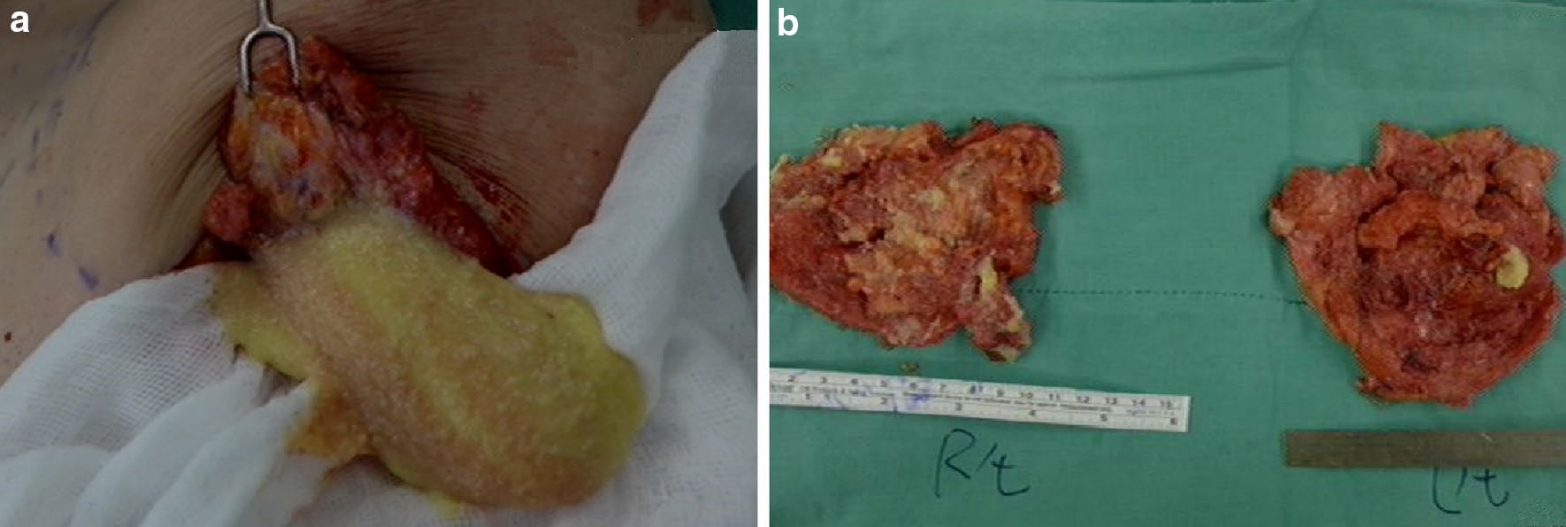
Intraoperative pictures. **a** The injected polyacrylamide hydrogel flow out with yellowish jelly-like appearance. **b** Simple mastectomy was done, and the hydrogel was removed with the infiltrated capsule

**Fig. 2:**
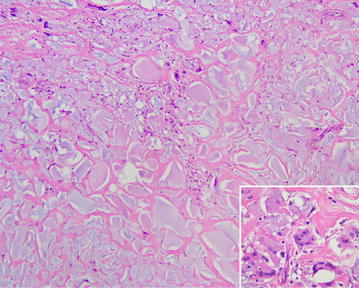
(HE stain, original magnification ×100, *right lower*: original magnification ×400). The breast shows variable sized gelatinous pools, which are surrounded by collagenous fibrous tissue. In some areas, foreign body reaction characterized by infiltrated foreign body type multinucleated giant cells and macrophages is seen (*right lower inset*)

**Fig. 3:**
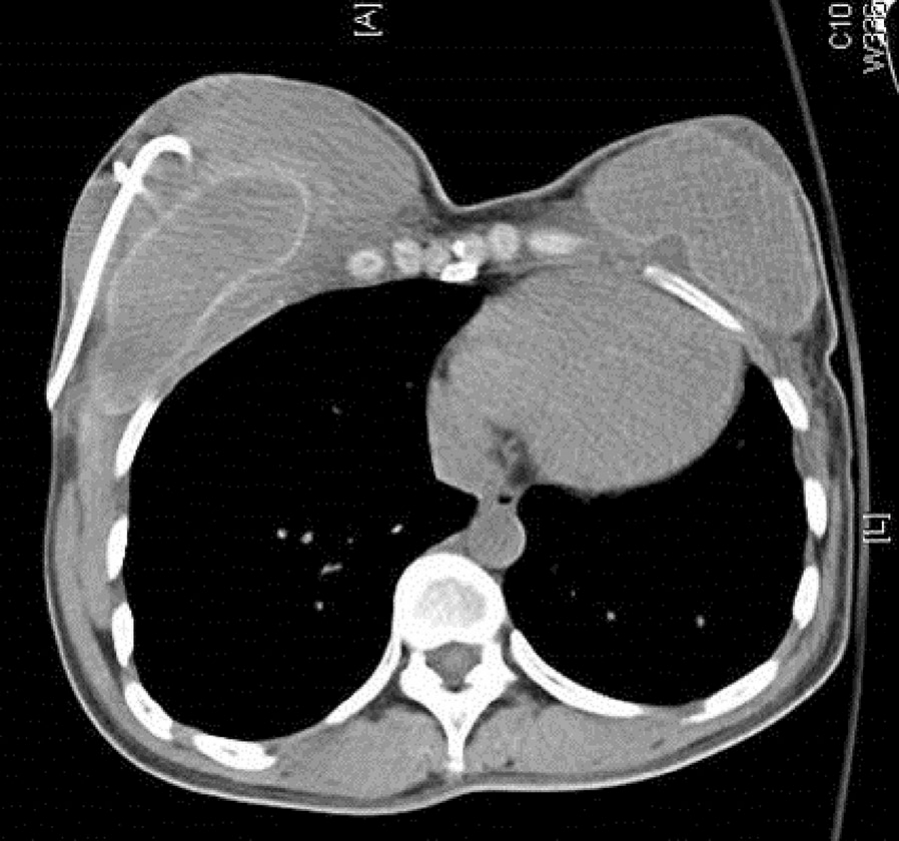
Computed tomography image of hematoma formation on the 14th day post simple mastectomy and immediate subpectoral silicon-implant reconstruction. Pigtail drainage tube was inserted and 250 ml hematoma was drained out

**Fig. 4:**
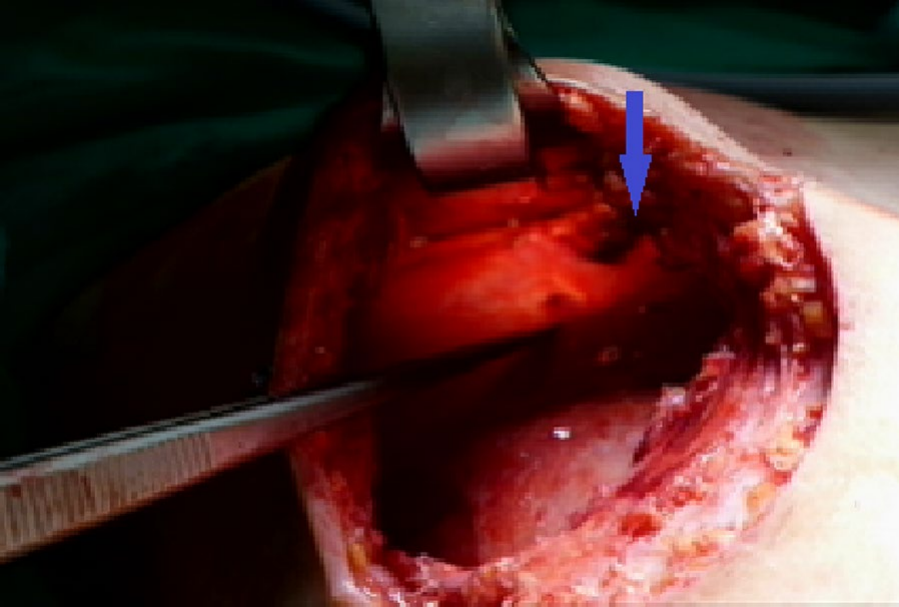
Right pectoral muscle oozing (*arrow*) over the 2nd intercostal space 2 cm paramidline to sternum was found during exploration surgery. Intercostal perforator bleeding was suspected

The 37-year-old G2P0A2 slim (BMI = 18.3) woman, who received injected PAAG bilateral breast augmentation in China 15 years ago, visited our hospital due to left upper quadrant breast tenderness with burning sensation lasting for 2 months. Breast ultrasound revealed architectural distortions of breasts. Bilateral simple mastectomy for radical evacuation of PAAG and immediate subpectoral smooth round silicon-implant reconstruction with Mentor MemoryGel™ were performed for symptoms relief as well as potential malignancy concern (Fig. 1). Pathologic report showed PAAG gel surrounded by collagenous fibrous tissue. Giant cells were also found, which implied a chronic inflammation process. There was no evidence of acute inflammation such as polymorphonuclear leukocytes infiltration (Fig. 2). The patient tolerated the procedure well and started breast massage on post-operation day14. However, her right breast swelled with tension immediately after massage, and she developed chest tightness and shortness of breath when she arrived at the emergency department. Subcutaneous hematoma of around 250 ml was drained by diagnostic computer tomography (CT) scan guidance (Fig. 3). For complete removal of the solitary residual hematoma, we did a secondary exploratory surgery where the lesion at the second intercostals space, 2 cm to the right of the sternum on the right pectoral muscle was noticed and coagulated (Fig. 4). The drain was removed smoothly 6 days after second operation. The patient was discharged uneventfully.

Written informed consent was obtained from the patient for the use of her medical information and images in the publication of this case report.

## Discussion

In 2010, Luo et al. reported a case series of 235 patients with PAAG-injected breast augmentation complication. The author concluded that the best solution for PAAG breast injection complication is the removal of PAAG gel and its infiltrated capsule (Luo et al. 2011). Open evacuation operation via periareolar incisions or inframammary fold approach (DmytroUnukovych et al. 2012; Yu et al. 2012) was suggested. A case series of 104 patients conducted by Yu et al. in 2012 suggested a preference of periareolar incisions over inframammary fold incision due the its advantage of better PAAG clearance rate (Yu et al. 2012). In addition, previous study showed that immediate reconstruction had less affective distress and better psychosocial outcomes in patients compared to delayed reconstruction (Al-Ghazal et al. 2000). Moreover, if breast contour restoration is requested by patients, immediate breast augmentation with silicone prostheses is suggested if there are no signs of acute inflammation. But for patient with acute inflammation, breast reconstruction is suggested to be delayed 5–6 months after PAAG gel removal to ensure that the inflammation process had subsided (Luo et al. 2011; Patlazhan et al. 2013).

In our patient, the concern of scar caused by chronic inflammation beneath the periareolar incision following nipple areola complex contracture led us to used inframammary fold incision. Due to her slim habitus with low breast fat content, and the borders of PAAG was hard to identify due to diffused infiltration, we performed a simple mastectomy to remove as much PAAG gel as possible and in the sake of further reconstruction of symmetry. Since there were no signs of acute inflammation or infection, immediate breast augmentation with silicone prostheses was carried out. However, subcutaneous hematoma formed right after breast massage.

Regarding the choice of breast implant for the breast reconstruction in patient who received PAAG removal, Patlazhan et al. reported a case series of 154 patients with capsule contracture rate of 24.8 % in texture surface implant reconstruction (Patlazhan et al. 2013). Since our patient was a PAAG injected slim patient with thin pectoral muscle and subcutaneous tissue, we used smooth round silicon-implant, which insures softer tactile impression and avoid rippling compare to the texture surface silicone implants. However, in such patients, texture surface breast implant may still have its advantages of less capsule contracture rate and no massage requirement, hence, prevent massage related surgical site bleeding. We also acknowledge the current data that when the implant was put under the pectoral muscle, the capsule contracture rate was actually similar between texture surface implant and smooth ones. The condition in patients who had received PAAG injected breast augmentation could be different because of the underlying pectoral muscle atrophy. However, to our understanding, there is no previous study comparing the effects of smooth and texture surface of breast implant on patients received PAAG removal.

Among literatures we have reviewed to date, reconstruction after evacuation of PAAG was mainly based on implant reconstruction. The role of lipo-filling and autologous tissues transfer in such patients hasn’t been put into consideration in current study yet. The uncertainty of skin circulation after PAAG evacuation could be the barrier for transferred fat survival. However, in patients with severe erosive muscle degeneration caused by PAAG injection, the application of muscle transferring flaps, such as Latissimus dorsi flap, to provide more tissue coverage for preventing subsequent capsule contracture could be a reasonable consideration.

To make a thorough inquiry of the reasons why our patient encountered such complication, several possibilities were assumed. First, the usage of PAAG could cause degeneration of muscle tissue structure through a chronic inflammation process, and makes it more fragile than normal muscle tissue (Leung et al. 2007). The pathologic report of this case illustrated PAAG surrounded by collagenous fibrous tissue with foreign body reaction characterized by multinucleated giant cells and macrophages, suggested a chronic inflammation process. Though there was no direct evidence of muscle atrophy identified in the pathology, there might still be some underlying erosive dysfunction under prolonged inflammatory process because muscle atrophy would not be taken note in the pathologic section unless severe structural damage had taken place. The patient’s breast ultrasound did reveal remarkable breast architectural distortion. We believed the ultrasound findings also supported our hypothesis of the existence of underlying erosive muscle degeneration. Secondly, our patient was slim, there might be lack of enough soft tissue coverage, cushion and muscle tissue protection which could cause bleeding by external compression or shearing while massage. It could also explain the reason why our patient’s bleeder appeared at the intercostal region where the vascular perforators emerge through. Intercostal muscle torn with perforator injury could lead to acute massive bleeding followed by restrictive lung expansion with symptoms such as chest tightness and shortness of breath.

For slim patients, we suggest that the postoperative breast massage should be postponed until the tissue totally recovered. The healthcare team should inform patients about the possibility of delayed surgical site active bleeding and the collision of breasts should be cautioned. In addition, although immediate breast reconstruction shown to have more benefits than delayed reconstruction in its feature of better psychosocial outcomes (Al-Ghazal et al. 2000), delayed breast reconstruction could still be considered in such a slim patient to preclude the potential risk of delay bleeding after immediate reconstruction even if there were no acute inflammation.

## Conclusion

The PAAG breast augmentation can cause mastopathy and even erosive muscle degeneration. Delayed surgical site active bleeding should be cautioned for slim patients who received implant-based breast reconstruction post radical evacuation with mastectomy when they started massage. We suggest that the postoperative breast massage should be postponed until the tissue recovered, or delayed breast reconstruction could be considered in such patient even though there is no acute infection in process. We are not able to make conclusion toward the selection of breast implants and the casual relationship to the delayed bleeding in our patient. In addition, we put forward the possible utility of muscle transferring flap in the reconstruction of patient received PAAG evacuation.
